# Lateral access mechanism of LPA receptor probed by molecular dynamics simulation

**DOI:** 10.1371/journal.pone.0263296

**Published:** 2022-02-03

**Authors:** Rieko Suenaga, Mizuki Takemoto, Asuka Inoue, Ryuichiro Ishitani, Osamu Nureki

**Affiliations:** 1 Department of Biophysics and Biochemistry, Graduate School of Science, The University of Tokyo, Bunkyo-ku, Tokyo, Japan; 2 Graduate School of Pharmaceutical Sciences, Tohoku University, Aramaki, Aoba-ku, Sendai, Miyagi, Japan; Universidade Nova de Lisboa Instituto de Tecnologia Quimica e Biologica, PORTUGAL

## Abstract

G-protein-coupled receptors (GPCR) are a family of membrane receptors that play important roles in the regulation of various physiological phenomena. LPA receptors (LPA_1-6_) are members of the class A GPCRs, which transduce a lysophosphatidic acid (LPA) signal across the cell membrane and evoke various responses, including cellular survival, proliferation, differentiation, and migration. The crystal structure of LPA_6_ revealed a gap between its transmembrane helices (TMs), which is opened toward the membrane side. This led to the proposal of the “lateral access model,” in which its lipophilic ligand directly enters the binding pocket through the gap structure at the membrane. In this study, we performed molecular dynamics (MD) simulations and Markov state model (MSM) analyses of LPA_6_ and LPA, to elucidate the long timescale dynamics of the ligand binding process. The results from the 71.4-μs MD simulation suggested that the flexibility of the TMs constituting the gap structure enables the lateral entrance of the ligand, and the key interactions between the receptor and ligand facilitate the transition state of the ligand binding process.

## Introduction

G-protein-coupled receptors (GPCRs) are a family of membrane receptors with a conserved motif of seven transmembrane alpha helices, which transmit extracellular signals into the cell. Triggered by the binding of extracellular ligands, GPCRs activate intracellular heterotrimeric G proteins, thereby evoking downstream signaling cascades. The ligands of GPCRs include amines, peptides, nucleic acids, and lipids, which play essential roles in the regulation of various physiological phenomena. In addition to their importance, their properties as receptors on cellular membranes make them promising drug targets.

**Fig 1:**
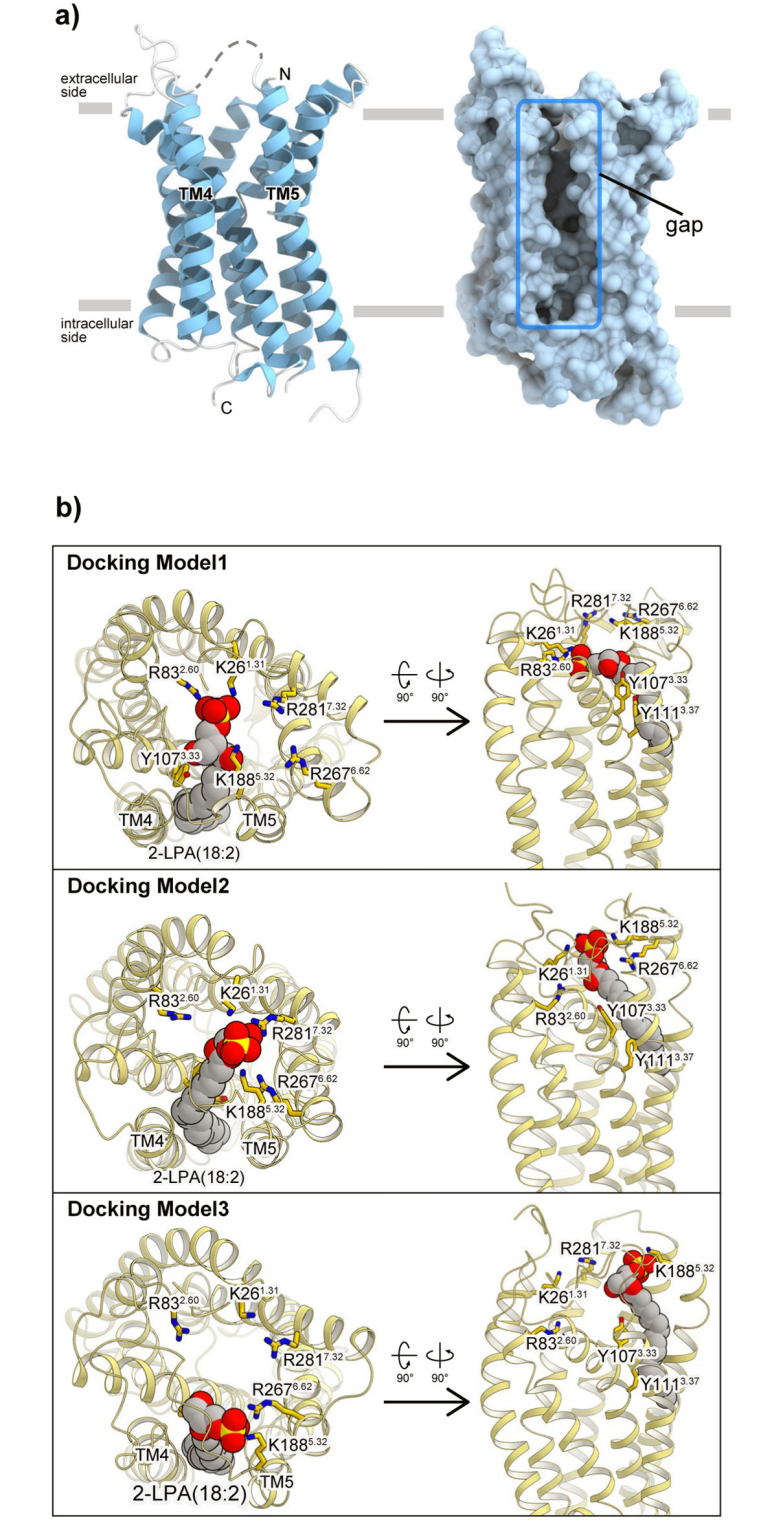
Structure of LPA_6_ and LPA_6_-LPA docking model. a) Overall crystal structure of zebrafish LPA_6_ (Protein Data Bank (PDB) ID: 5XSZ), viewed from the membrane plane. In the left panel, the whole structure is shown as a ribbon model, and the disordered region of ECL2 is illustrated as a dashed line. In the right panel, the whole structure is shown as a surface model from the same viewpoint as the left panel. The gap structure between TM4 and TM5 is indicated with the blue rectangle. b) The LPA_6_-LPA docking models (Models 1, 2, and 3) viewed from the extracellular side (left), and the membrane plane (right). The protein is shown as a ribbon model and the LPA (2-LPA(18:2)) molecule is depicted by a CPK model. The conserved positively charged residues are shown as stick models.

Lysophosphatidic acid (LPA) is a lipid mediator that induces various cellular responses, including survival, proliferation, differentiation, and migration [1]. The LPA receptors form a group of class A GPCRs, including LPA_1-6_. Among these receptors, LPA_6_ is reportedly important for hair formation [2, 3] and cancer progression [4], and thus regarded as a potential target for anti-cancer agents. Recently, our group reported the 3.2 Å crystal structure of LPA_6_ [5] (Fig 1a), which revealed a “gap” open toward the lipid bilayer between transmembrane helix (TM) 4 and TM5. A monoolein molecule was observed in this gap, and thus we proposed that the monoolein molecule binds to the gap by mimicking the acyl chain of LPA. In addition, based on the arrangements of the transmembrane helices and important acidic residues, we suggested that this crystal structure represents a pre-activation state, in which the receptor is bound to LPA, but has not adopted the active conformation. The lipophilic LPA may laterally access the ligand binding pocket from the side of the receptor, by passing through this gap. We also proposed some models of the LPA-LPA_6_ complex in the pre-activation state, based on the docking simulation [5]. Similar gap structures have also been observed in other lipid-accepting GPCRs, including sphingosine 1-phosphate receptor 1 (S1P1) [6], cannabinoid receptor 1 (CB1) [7, 8], thromboxane A2 receptor (TP) [9], prostaglandin E receptor 4 (EP4) [10], prostaglandin D2 receptor (DP2) [11], human GPR40 receptor (hGPR40), also known as free fatty-acid receptor 1 (FFAR1) [12, 13], and platelet-activating-factor receptor (PAFR) [14]. Therefore, the lateral access through a gap structure might be a common mechanism among GPCRs for lipophilic ligands.

Although it is difficult to experimentally verify the lateral access mechanism of lipophilic ligands from the viewpoint of structural biology, molecular dynamics (MD) simulations can provide important insights into the ligand binding process. In this study, we performed a series of all-atom molecular dynamics simulations of the ligand binding process of LPA_6_. Since the timescale of the ligand binding process may exceed those accessible by the conventional MD simulation method, we utilized the Markov state model (MSM) analysis [15] to capture the long timescale and rare events. MSM describes molecular kinetics as memoryless probabilistic transitions between a set of conformational states, and has been successfully applied to solve several problems, including protein folding [16], the activation mechanism of the β2-adrenergic receptor [17], and the ligand binding process of a soluble protein [18]. Our present results, reconstructed from the 71.4 μs MD simulations, successfully visualized the LPA binding process. Furthermore, the results provided detailed insights into the lateral access mechanism of the lipophilic ligand, including the transition state formation and the mechanism of ligand entrance into the binding pocket of LPA_6_.

## Results

### Markov state model of the ligand binding process

To reconstruct the long timescale dynamics of the ligand binding pathway, we tried to build a Markov State Model (MSM) from multiple short MD simulations [15]. MSM construction requires a sufficient number of short MD simulations, starting from initial states and covering from the ligand-unbound state to the ligand-bound state. In our previous study, we obtained three LPA_6_ ligand binding models by a docking simulation [5] (Fig 1b). Firstly, we analyzed three docking models (Models 1, 2, and 3). In Models 1 and 2, the LPA head group is deeply accomodated in the binding pocket and interacts with residues on TM1, 2, and 7. In contrast, in Model 3, the head group only interacts with the residues on TM5 and 6, which are exposed on the extracellular side. As a result, the interactions between the LPA head group and the basic residues of the receptor are different. In Model 1, the head group is bound to three residues, K26^1.31^, R83^2.60^ and K188^5.32^. In Model 2, the head group contacts K26^1.31^, K188^5.32^, R267^6.62^ and R281^7.32^, and in Model 3, the head group is only bound to K188^5.32^ and R267^6.62^. While our previous functional analysis showed that K26^1.31^, R83^2.60^ and R281^7.32^ are important for LPA_6_ activation [5], the interactions with these residues are absent in Model 3. Therefore, we used Models 1 and 2 as the initial structures of the simulation.

**Fig 2:**
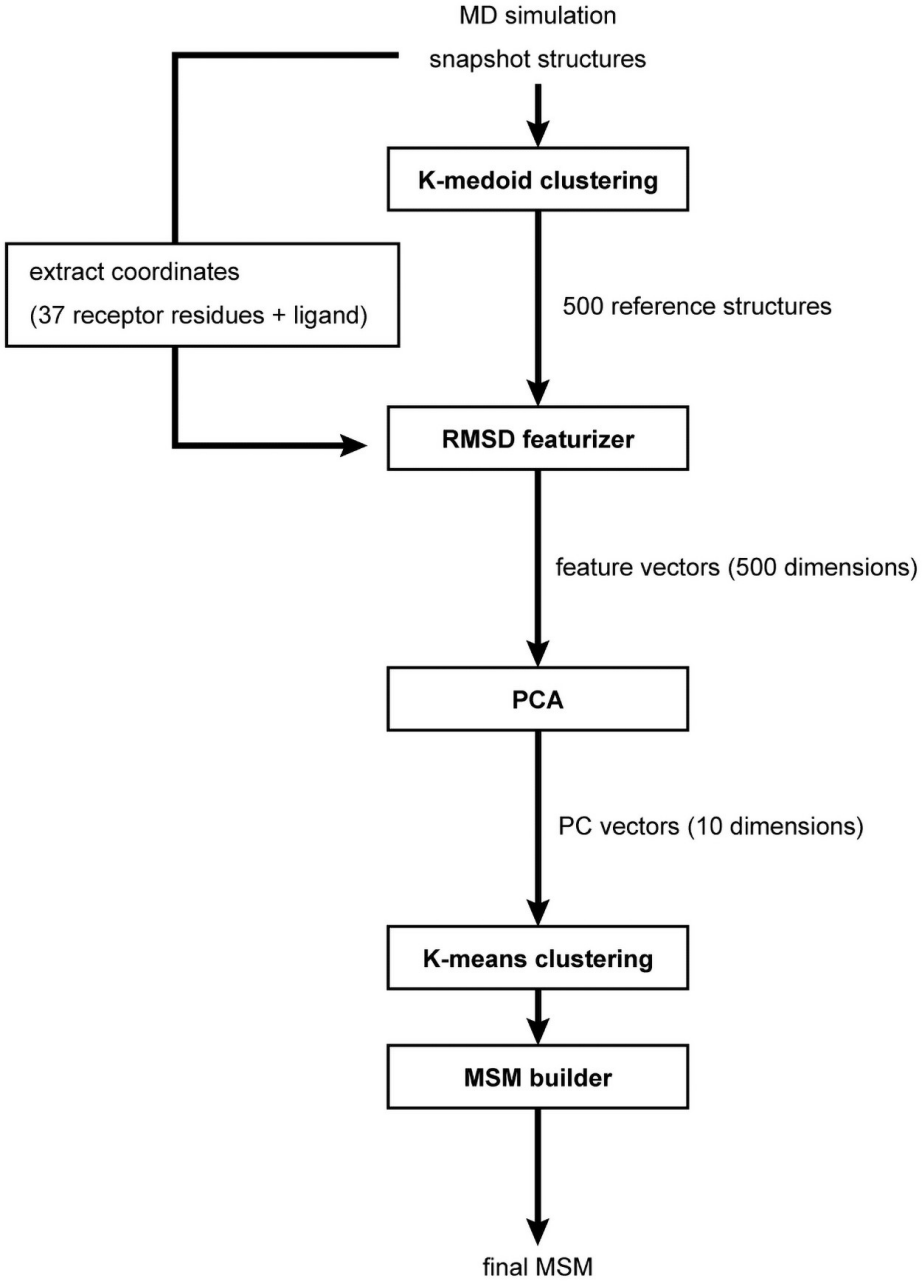
Schematic flow-chart of the procedure used in this work for MSM construction.

We next obtained the sequential trajectory from the ligand-bound state to the unbound state by a “steered MD” simulation, in which the ligand was slowly separated from the receptor with external force. We then conducted non-biased MD simulations starting from the 379 and 335 snapshot structures of the steered MD simulations of Models 1 and 2, respectively. Finally, we collected the 37.9 μs and 33.5 μs MD trajectories for Models 1 and 2, respectively. Although the trajectories from Model 1 and Model 2 were independently analyzed, we hereafter discuss the results from Model 1, unless otherwise noted, since the results from Models 1 and 2 share numerous similarities (S2 Fig). To build the MSM for these simulations, we analyzed the MD simulation trajectories, as shown in Fig 2. The RMSD values to the “reference structures” were calculated using the ligand non-hydrogen atoms and protein atoms interacting with the ligand, thereby forming the feature vectors. These feature vectors were then compressed using the principal component analysis (PCA), and were finally subjected to the clustering and MSM analysis. The robustness of the analysis was verified by the bootstrap method (S1 Fig). Detailed descriptions of the simulation and MSM construction are provided in the S1 File.

### Definition of macrostates

**Fig 3:**
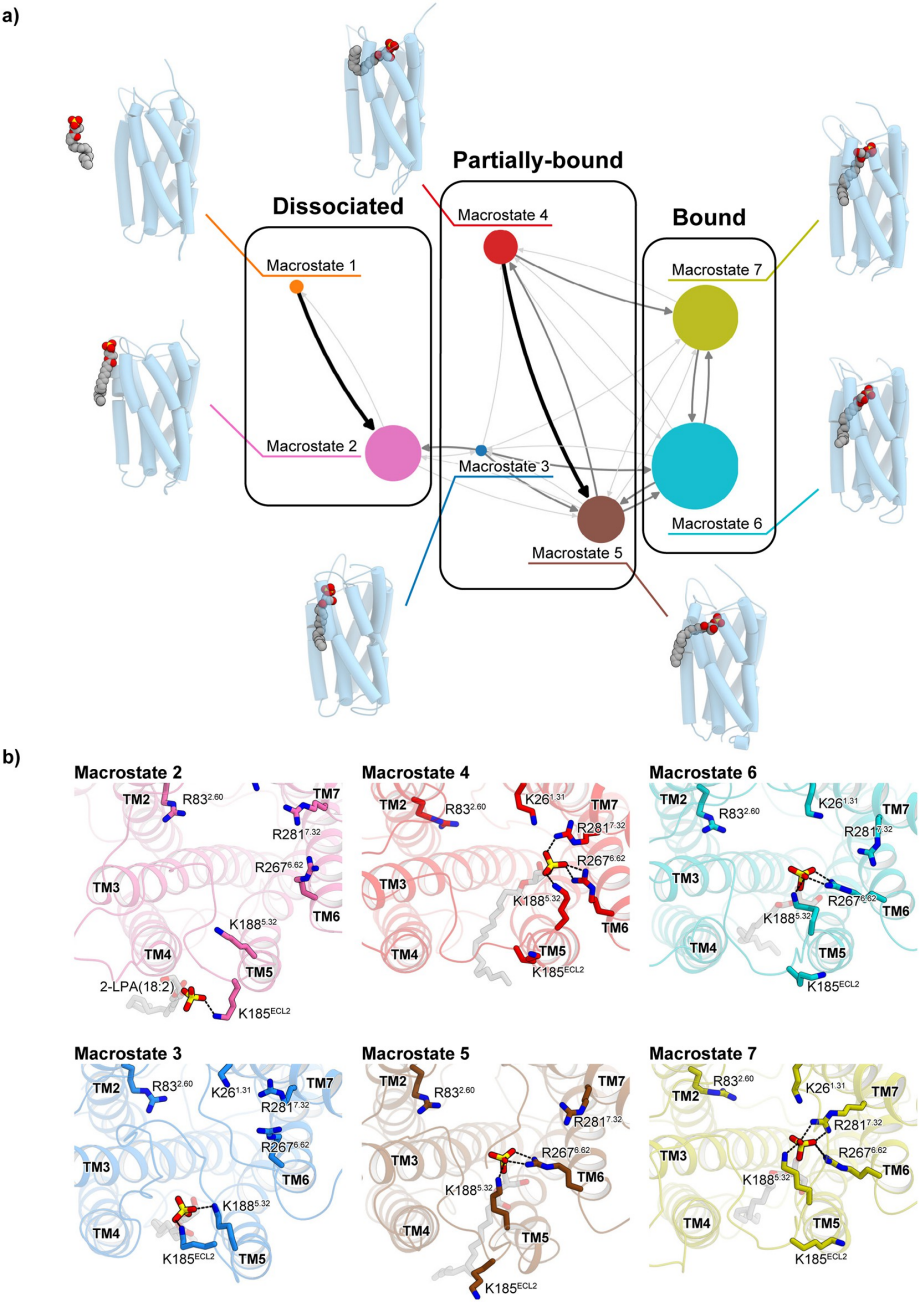
Macrostates and MSM groups calculated from the present simulation results. a) The macrostates observed in the present simulation. The size of the circle corresponds to the population of the microstate. The transitions between the macrostates are indicated by the gray arrows. The thickness of the arrows is proportional to the transition probability. The structures of the centers of the largest clusters are shown with cartoon and CPK models. b) Close-up views of representative structures of the macrostates. The interactions of the ligand-ligand recognition residues in each structure are shown as ball and stick models.

We classified the 995 microstates into seven “macrostates” by the PCCA+ method [19]. The microstate structure with the largest population in each microstate was visualized as its representative structure (Fig 3a). These macrostates were then divided into three groups (i.e., dissociated, partially-bound, and bound groups).

The ligand-unbound dissociated group was classified into two macrostates, Macrostates 1 and 2 (Fig 3a). In Macrostate 2, the ligand molecule was adjacent to the receptor gap. Particularly, in its representative structure, the ligand head group was trapped by the positive residue K185 on extracellular loop 2 (ECL2) (Fig 3b).

In the partially-bound group, which includes Macrostates 3, 4, and 5, the ligand was partially bound within the receptor gap, and a small number of interactions were formed. In Macrostate 3, the ligand head group was only bound to K185^ECL2^ and K188^5.32^, and did not interact with any functionally important basic residues [5]. In Macrostates 4 and 5, the ligand head group formed interactions with R267^6.62^, but the acyl chain was not fully accommodated within the receptor gap (Fig 3b). Therefore, this group may include transition states between the ligand-bound and -unbound states.

In the bound group, which includes Macrostates 6 and 7, the ligand was fully accommodated by the receptor and formed interactions with the functionally important basic residues (Fig 3a). In Macrostate 7, the ligand formed the deepest interactions with the receptor, and the head group was bound to K188^5.32^, R267^6.62^ and R281^7.32^, whereas it was bound to only K188^5.32^ and R267^6.62^ in Macrostate 6 (Fig 3b). In the following sections, we will discuss these seven representative structures.

### Energy landscape and transition path analysis of the ligand binding process

**Fig 4:**
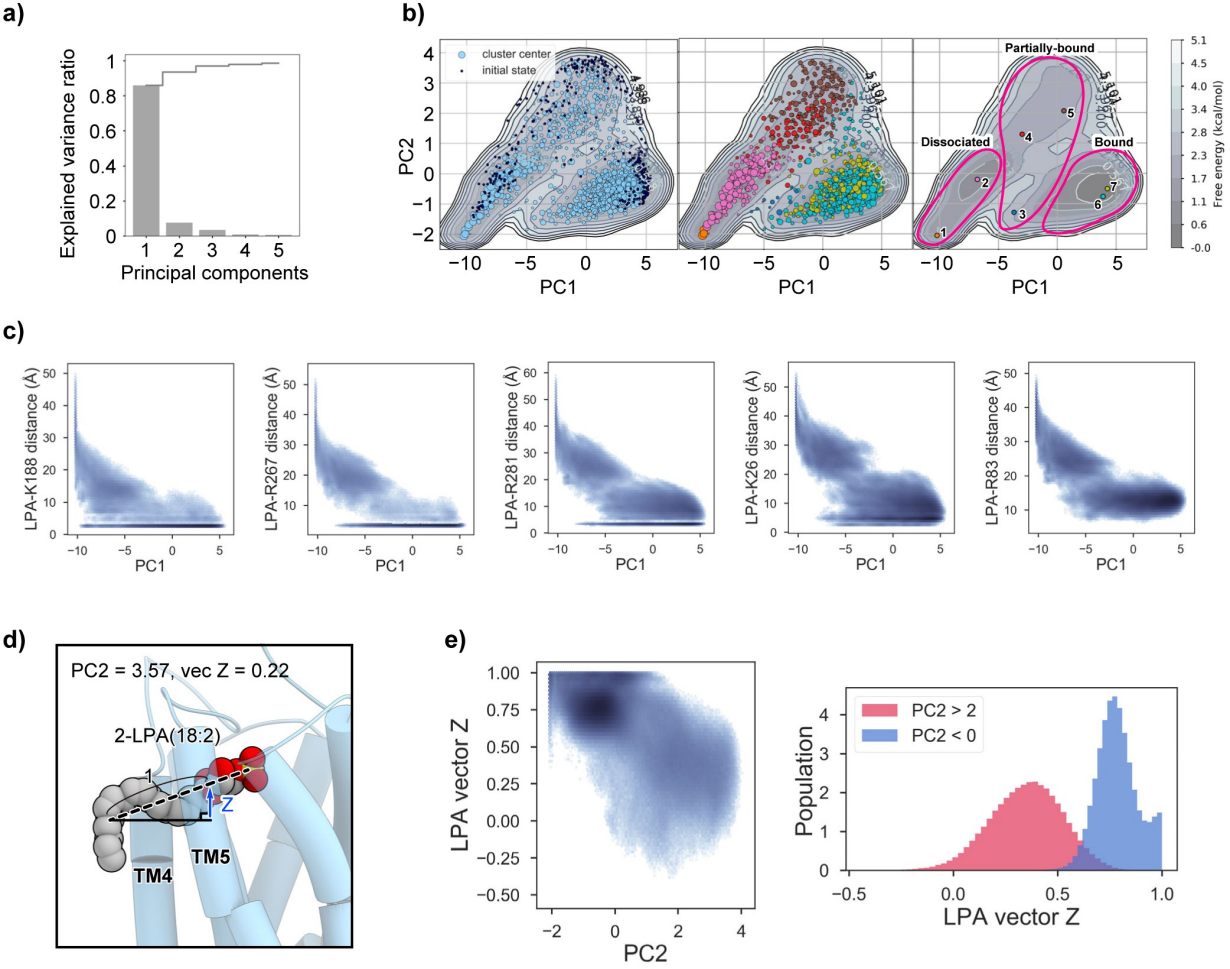
Free energy landscape projected on the plane spanned by the PC1 and PC2 axes. a) The explained variance ratios of PC1 to PC5 (gray bar chart), and their cumulative values (gray line). b) Free energy landscape projected on the PC1-PC2 plane. In the left panel, the centers of the clusters and the initial structures are plotted as light and dark blue circles, respectively. The sizes of the light blue circles are proportional to the populations of the clusters. In the middle panel, the centers of the clusters are color-coded according to their macrostates. In the right panel, the center of each microstate is plotted on the free energy map. The groups of the macrostates defined in Fig 3 and the main text (i.e., dissociated, partially-bound, and bound groups) are indicated by magenta lines. c) The relationships between the PC1 values and the distances between the LPA head group and the conserved positive residues (K188^5.32^, R267^6.62^, R281^7.32^, K26^1.31^, and R83^2.60^). The distances are defined as those between the nearest oxygen atom of the head group and the nitrogen atom of the basic residues. d) The ligand conformation and the receptor structure with the highest PC2 value (PC2 = 3.57). The ligand and receptor are shown as CPK and cartoon models, respectively. The definition of the ligand vector and its z value are depicted as the dashed line and blue arrow, respectively. e) The relationship between the PC2 value and the z value of the ligand shown in (d). The difference of the distributions of the ligand z values in the higher and lower PC2 regions (red and blue for PC2 > 0 and < 2, respectively) is shown as a histogram in the right panel.

We calculated the microstate population distribution in the equilibrium state from the MSM, and then estimated the free energy landscape by projecting it onto the two-dimensional plane spanned by the PC axes obtained by the PCA of the 500-dimensional feature vectors (Fig 4). In the PCA, the cumulative variance ratio of the PCs was over 97% from PC1 to 3 (Fig 4a), showing that the ligand binding process can be explained by these three PCs. Thus, we hereafter focused on them.

We first projected the microstate population distribution on the plane spanned by the PC1 and 2 axes, and plotted the cluster centers on it (Fig 4b). The plot revealed two low-energy basins at the low- and high-PC1 regions, which may correspond to stable states in the ligand binding process. The detailed analysis suggested that PC1 is simply related to the ligand-receptor distance, with high and low PC1 values corresponding to dissociated and bound states, respectively. In contrast, PC2 is correlated with the relaxation process after removing the biasing potential of steered MD, with high and low PC2 values corresponding to the biased and relaxed states, respectively (see S1 File). The low free-energy basins around the low PC2 regions suggested that the sampling of the relaxed conformations was sufficient to provide an unbiased view of the ligand binding dynamics.

**Fig 5:**
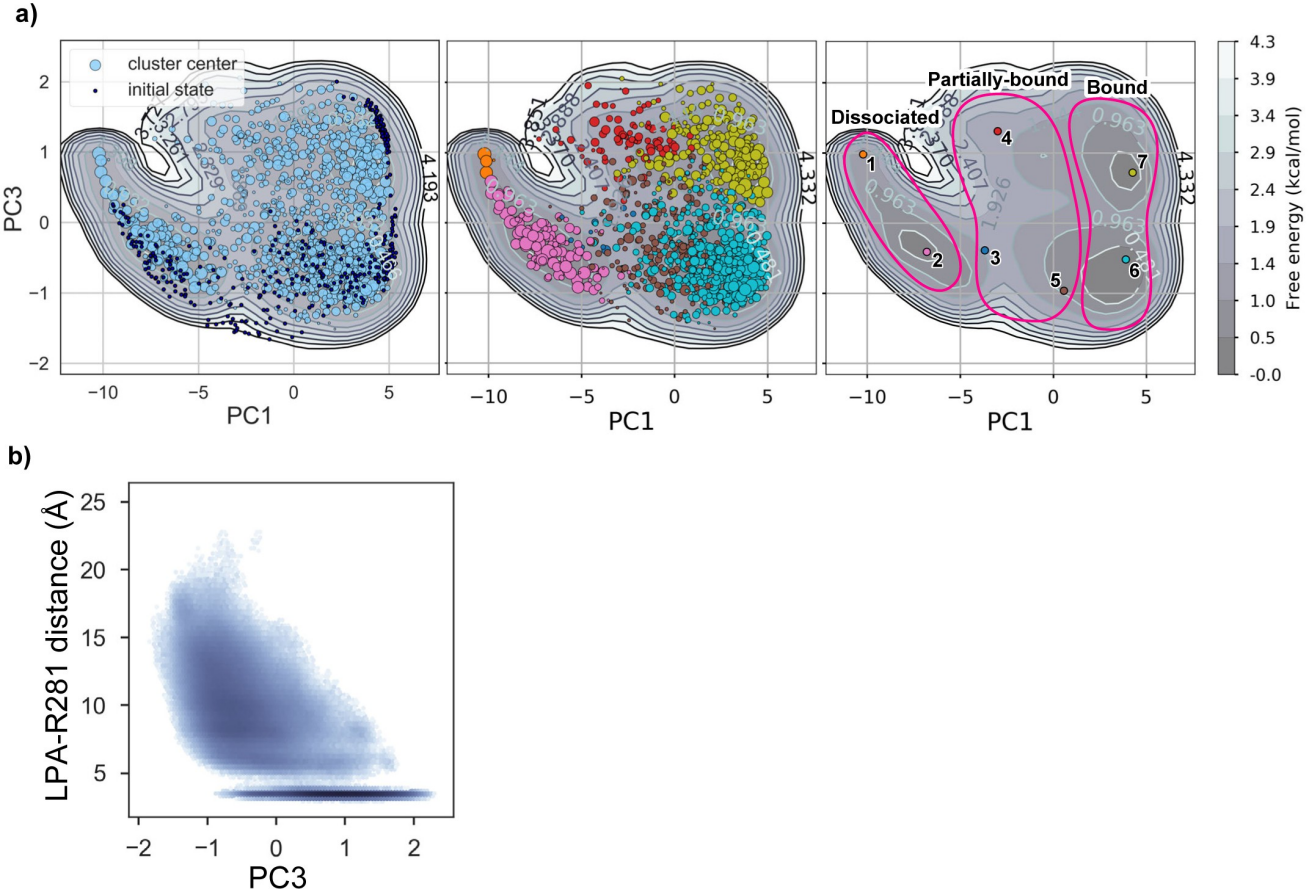
Free energy landscape projected on the plane spanned by the PC1 and PC3 axes. a) Free energy map projected on the PC1-PC3 plane. In the left panel, the centers of the clusters and the initial structures are plotted as light and dark blue circles, respectively. The sizes of the light blue circles are proportional to the populations of the clusters. In the middle panel, the centers of the clusters are color-coded according to their microstate. In the right panel, the positions of the central structures of the macrostates are indicated by circles. A basin in the free energy map with a small PC1 value corresponds to a dissociated state, that with an intermediate PC1 value represents a partially-bound state to the central region with high energy, and that with a large PC1 value corresponds to a bound state. Macrostate 6 corresponds to a basin with a low PC3 value, and 7 corresponds to a basin with a high PC3 value. b) Relationship between the PC3 value and the distance between the LPA head group and R281^7.32^. The definition of the distance is the same as in Fig 4.

We next projected the probability distribution on the plane spanned by the PC1 and 3 axes, and plotted the cluster centers (Fig 5). In the PC1-3 plane, the basin around the “bound” group in the PC1-2 plane was separated into two basins, the low-PC3 and high-PC3 basins, which correspond to Macrostates 6 and 7, respectively (Fig 5a). The detailed analysis suggested that the PC3 axis corresponds to the process for deeply accommodating the ligand head group within the basic pocket formed around R281^7.32^ (see S1 File).

### Reconstruction of the ligand binding/unbinding trajectory

**Fig 6:**
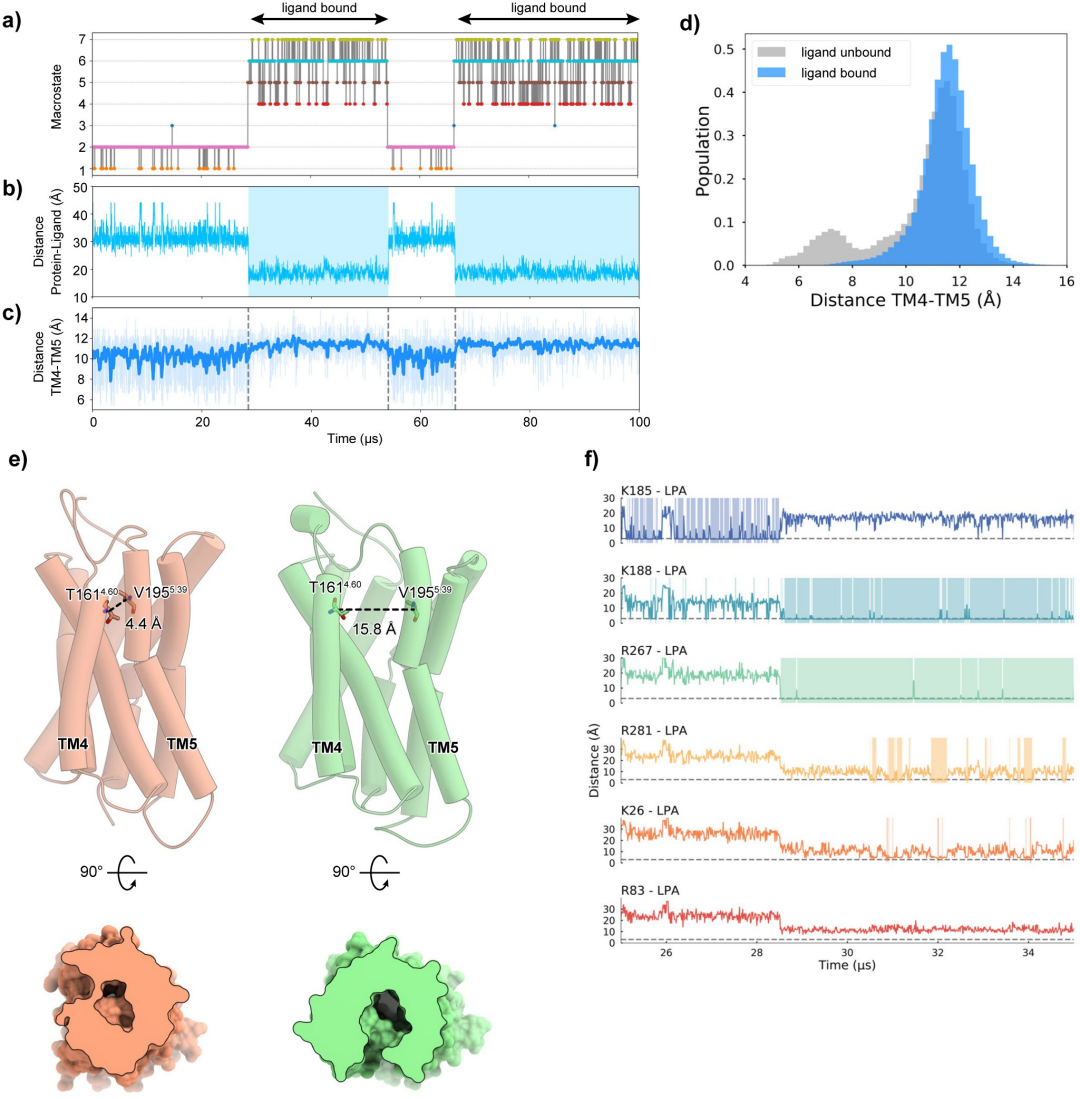
Reconstituted 100-μs trajectory of the LPA binding process. a) Plot of the transitions among Macrostates 1–6 of the reconstituted trajectory. The color code corresponds to that used in Fig 3. b) Plot of the transition of the distance between the LPA head group and the center of mass of the receptor protein. Distances less than 25 Å are highlighted in light blue. c) The transition of the TM4-TM5 distance is plotted in light blue, and the moving average between the four frames is plotted in blue. d) The distribution of distances between TM4 and TM5 in the ligand-unbound (gray) and -bound (blue) states. e) The structures with the smallest (orange) and largest (green) TM4-TM5 distances in the ligand-unbound state are shown by cartoon models and cross-sectional views of the electron density surfaces. f) Plot of the distances between the LPA head group and the conserved positive residues (K185, K188^5.32^, R267^6.62^, R281^7.32^, K26^1.31^, and R83^2.60^) around 25 μs and 35 μs. Areas with distances of 2.5 Å or less are highlighted in light blue and light green backgrounds.

Using the MSM, we reconstructed a 100-μs trajectory starting from the ligand-unbound state (Fig 6, S1 Movie). First, we analyzed the transitions among the macrostates (Fig 6a), and then the LPA binding to the receptor, by measuring the distance between the phosphate group of LPA and the center of the receptor (Fig 6b). The results showed that the reconstructed trajectory includes both the ligand binding and dissociation events, which occur on the order of 10 μs. Our previous structural analysis of LPA_6_ suggested that the membrane-embedded LPA molecule laterally enters the binding pocket, through the gap formed between TM4 and TM5 (TM4-5 gap) [5]. To investigate the dynamics of the TM4-5 gap, we measured the distance between the Cα atoms of T161 and V195. In the ligand-unbound state, the width of the TM4-5 gap fluctuated from 4.4 Å to 15.8 Å (Fig 6c), revealing its flexibility. In the snapshot structure with the smallest gap width, the TM4-5 gap is completely closed, and the ligand binding site is occluded from the lipid membrane (Fig 6e). In contrast, in the ligand-bound state, the fluctuations of the width of the TM4-5 gap are smaller than those in the ligand-unbound state. The minimum width was 6.9 Å, which is slightly wider than those of the ligand-unbound state (Fig 6c). The gap width histogram also revealed the differences in the flexibility between the ligand-bound and -unbound states (Fig 6d). In the transition state between the ligand-bound and -unbound states, in which the head group of LPA enters the pocket of the receptor, the width of the TM4-5 gap was about 12 Å.

We next analyzed the interactions between the ligand and the binding pocket residues, focusing on the ligand binding process observed around 30 μs in the reconstituted trajectory (Fig 6f). In the trajectory, the ligand head group initially formed a weak interaction with K185 on the ECL2 loop, as observed in the representative structure of Macrostate 2 (Fig 3b). Simultaneously, the head group also formed an interaction with K188^5.32^; however, its frequency was lower than that of K185. This stage (25–28.5 μs) mainly corresponds to the dissociated group (Macrostates 1 and 2) in the macrostate clusters. The head group then enters the TM4-5 gap, and forms interactions with K188^5.32^ and R267^6.62^. The interaction with R267^6.62^ is particularly stable and maintained throughout the ligand-bound state, suggesting its importance for the first step in the ligand binding process. In contrast, the interaction between the acyl chain and the TM4-5 gap has not been stably formed yet. Next, at 1.5 μs after binding with R267^6.62^, the head group is deeply accommodated within the pocket and interacts with R281^7.32^ and K26^1.31^. The interaction with R281^7.32^ induces a slight inward shift of the extracellular side of TM7. The acyl chain of LPA is also accommodated in the TM4-5 gap. This stage corresponds to the bound group (Macrostates 6 and 7) of the macrostate clusters. In contrast, we did not observe any interactions between the head group and R83^2.60^, which were observed in the initial docking pose of Model 1 (Fig 1b). Thus, the resulting bound state of the reconstituted trajectory is similar to Model 2, in which no interactions are formed between the head group and R83^2.60^.

**Fig 7:**
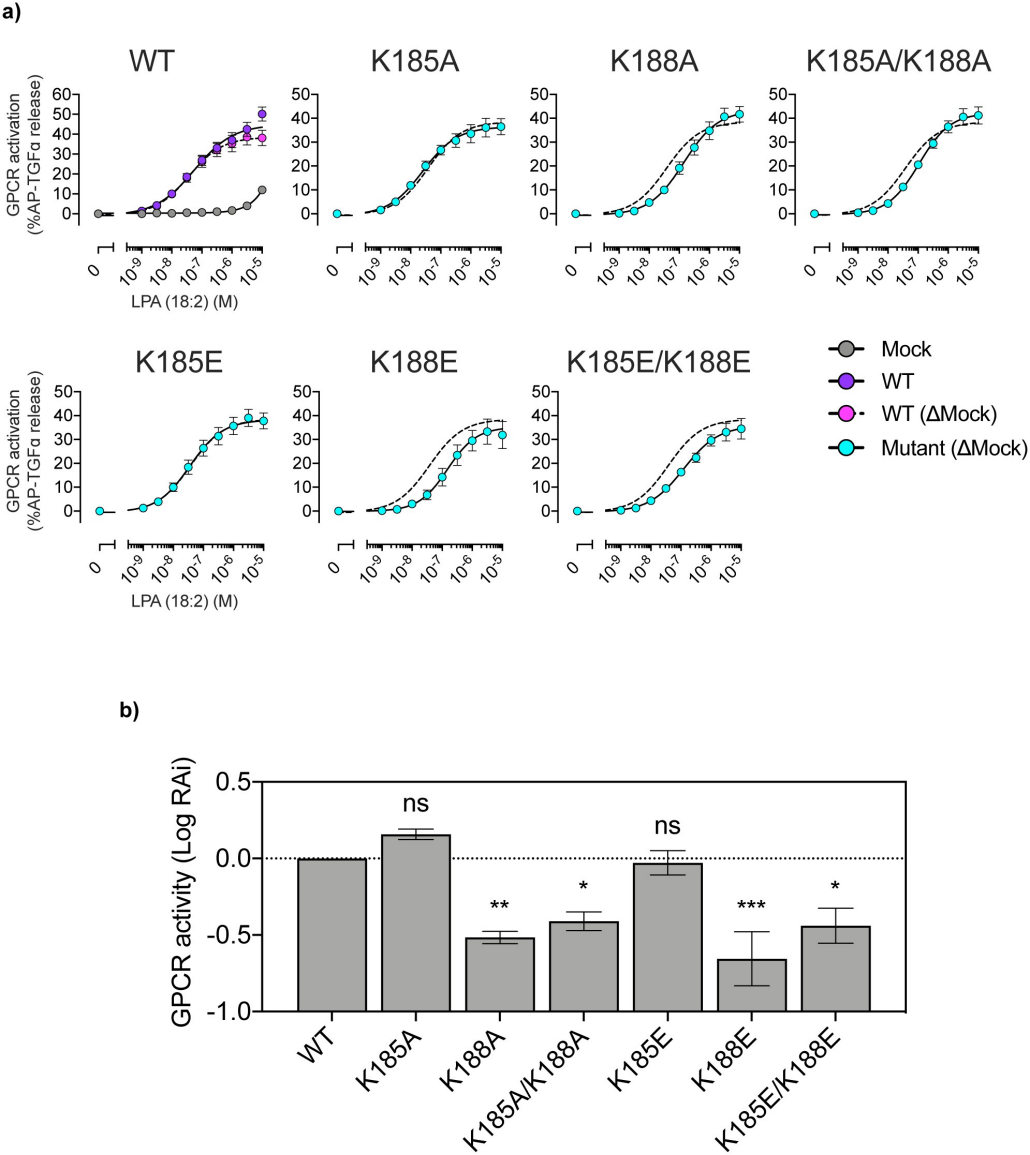
Functional analysis of LPA mutants designed based on the present study. a) The receptor activities were examined using the alkaline-phosphatase tagged TGF-ɑ shedding assay. b) Activities of LPA and its mutants expressed as RAi (E_max_/EC_50_ relative to WT). Data are mean ± s.e.m. (n = 3 or 4). *P < 0.05; **P < 0.01; ***P < 0.001, one-way ANOVA with Dunnett’s post hoc test. NS, not significant.

The macrostates and their relationships (Fig 3), as well as the reconstructed trajectory (Fig 4), suggested that K185 on ECL2 is another key residue in the ligand binding process. K185 forms a salt bridge with the phosphate moiety of the ligand head group in Macrostates 2 and 3, before forming the transition state in Macrostate 5 (Fig 3b). Afterwards, it dissociates from the ligand in Macrostates 5, 6, and 7 (Fig 3b). Therefore, the present results suggest that the positive charge of K185 anchors the ligand head group, thereby facilitating the formation of the transition state in the ligand binding process. To examine the importance of K185, we measured the signal transduction activities of single mutants (K185A and K185E) and double mutants (K185A/K188A and K185E/K188E) of the zebrafish LPA_6_ receptor. However, none of these mutations had a large impact on the activity (Fig 7). The effect of anchoring by K185 may be less critical than that of the activation for signal transduction, which makes it difficult to confirm the contribution of K185 by the assay method used in this study. Further analyses are required to corroborate the role of K185 in the ligand anchoring.

## Discussion

In this study, we successfully reproduced the pathway from the ligand-unbound state to the pre-activation state by MSM, based on the all-atom MD simulation. Our results support the lateral access model of the LPA ligand, and provide further detailed insights into the mechanism of ligand access to the LPA_6_ receptor. Particularly, the present simulation results suggest the importance of the flexibility of the TM4-5 gap, which forms the entrance of the receptor pocket. In the course of the simulation, the TM4-5 gap spontaneously opens and closes, allowing the ligand to enter the pocket of the receptor. The width distribution of the TM4-5 gap structure ranges from 8 to 12 Å. After ligand binding, the TM4-5 gap is stabilized in its open conformation by interactions with the ligand. Similar conformational changes in the gap have been reported for other lipid mediator GPCRs. For example, cysteinyl leukotriene receptor 1 (CysLT1R) also has a gap between TM4-5, which is similar to that of LPA_6_ [20]. The 1-μs MD simulation of CysLT1R demonstrated that the transition between the gap-open and gap-closed conformations spontaneously occurred, suggesting the relatively low energy barrier between them [20]. Furthermore, the binding of the membrane lipid molecule (POPC) reportedly stabilizes the gap in its open conformation [20]. The importance of the gap flexibility has also been mentioned for cannabinoid receptor 1 (CB1), although unlike LPA_6_, the gap is between TM1 and TM7, based on the comparison of the agonist- and antagonist-bound crystal structures, as well as the MD simulation results [21]. The MD simulations of CB1 also suggested that the binding of POPC in the gap stabilizes it in the open conformation [21]. Thus, the flexibility of the TM gap may be an important feature of GPCRs with lipophilic ligands, across the differences in the gap-forming TMs. It is interesting to note that the crystal structure of LPA_6_ was determined without an agonist or antagonist, while all of the other GPCRs with the gap structure were determined in the agonist- or antagonist-bound states [5]. In the crystal structure of LPA_6_, the monoolein molecule was observed in its TM4-5 gap, and may have stabilized the gap structure for crystallization.

**Fig 8:**
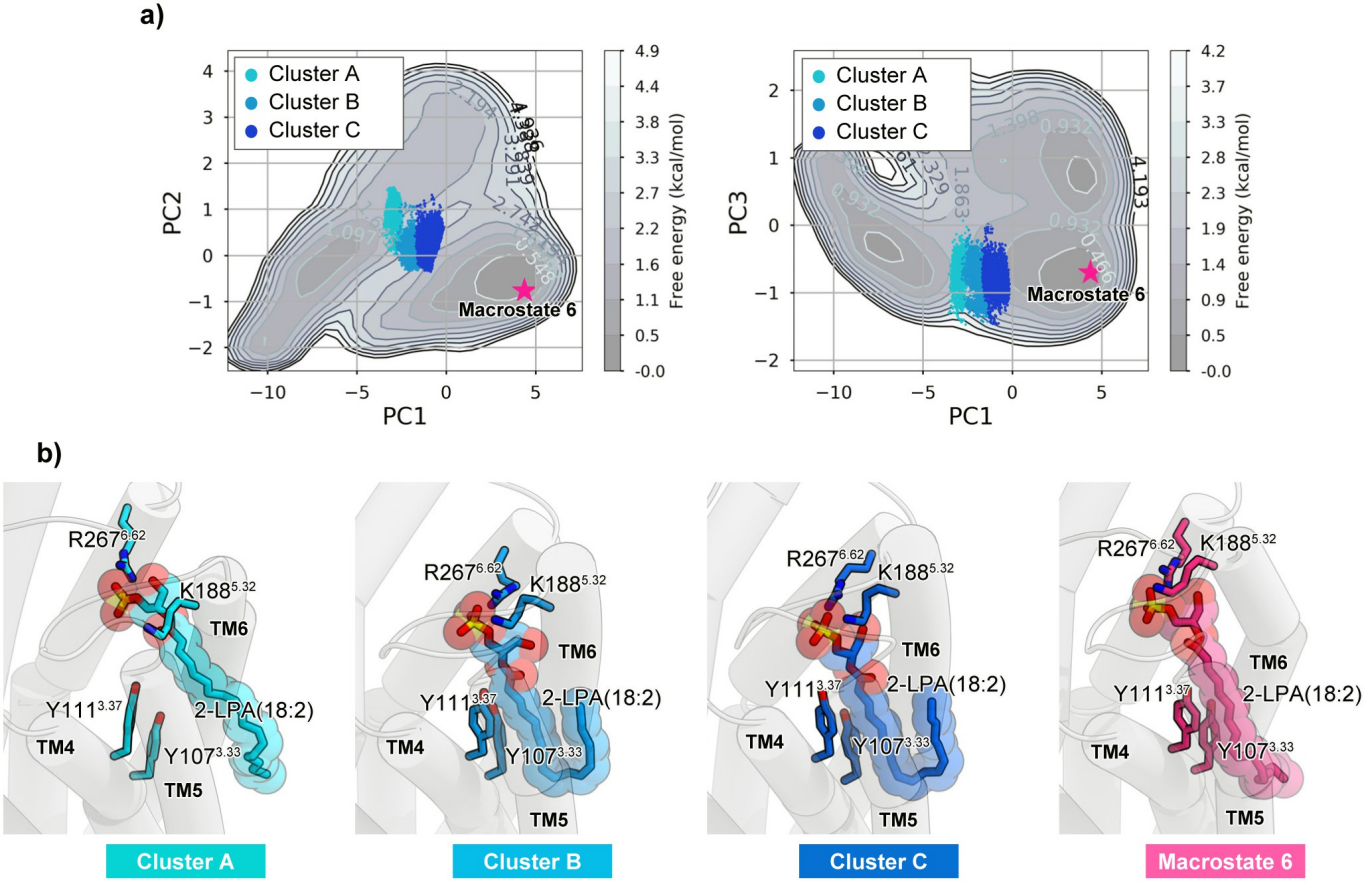
Transition state of the ligand binding process. a) The structures contained in the three clusters corresponding to the transition states, clusters A, B and C, are plotted on the PC1-PC2 and PC1-PC3 planes, respectively. The central structure of Macrostate 6 is indicated by pink stars. b) The central structures of clusters A, B and C, and the central structure of Macrostate 6 are shown by cartoon and ball and stick models, respectively.

The present simulation provides novel insight into the ligand binding process. The transition state in the binding process is particularly interesting, and can be analyzed from the energy landscape calculated in the present simulation. The transition state may correspond to the state between the unbound- and bound-states with the lowest energy barrier. The energy landscape projected onto PC axes 1–3 suggests that such a saddle point in the energy landscape may exist around the region with PC1 of −5 ~ 0, PC2 of 0 ~ 1, and PC3 of −1 ~ 0. Thus, we selected the three largest clusters from the region (clusters A, B, and C), and compared their representative structures (Fig 8a). Given that i) the direction of the PC1 increase corresponds to the ligand binding process, and ii) the PC1 value increases in the order of A, B, and C, we can assume that the conformational change toward the LPA binding occurs in this order. All of these clusters belong to Macrostate 5 (Fig 5a), which is one of the partially-bound groups. In clusters A, B, and C, the head group of LPA is accommodated in the binding pocket and forms interactions with K188^5.32^ and R267^6.62^ (Fig 8b). In contrast, the acyl chain tail of LPA lacks interactions with the receptor in cluster A, and instead partially interacts with the TM4-5 gap in clusters B and C (Fig 8b). The acyl chain tail interacts with the two aromatic residues Y107 and Y111 on TM3, and its terminus is still unstable due to the lack of interactions with the receptor (Fig 8b). Thus, after the head group binds, the acyl chain tail may first contact Y107 and Y111, thereby forming the binding transition state. After the terminus of the acyl chain tail is accommodated in the TM4-5 gap, the head group may be pushed deeply into the binding pocket to form the pre-activation state (Macrostate 6; Fig 8b). Taken together, the present simulations suggest that Y107 and Y111, which form part of the TM4-5 gap, could play a key role in the transition state formation in the ligand binding process. In addition, these aromatic residues are well conserved not only in phylogenetically-related GPCRs, such as P2Y15 and P2Y126, but also in PAFR7 and CysLT1R2, possessing the TM4-5 gap. Accordingly, the ligand binding process and transition mechanism might be conserved in these GPCRs.

The macrostates and their relationships (Fig 3), as well as the reconstructed trajectory (Fig 4), suggested that K185 on ECL2 is another key residue in the ligand binding process. K185 forms a salt bridge with the phosphate moiety of the ligand head group in Macrostates 2 and 3, before forming the transition state in Macrostate 5 (Fig 3b). Afterwards, it dissociates from the ligand in Macrostates 5, 6, and 7 (Fig 3b). Therefore, the present results suggest that the positive charge of K185 anchors the ligand head group, thereby facilitating the formation of the transition state in the ligand binding process. To examine the importance of K185, we measured the signal transduction activities of the single mutants (K185A and K185E), as well as the double mutants (K185A/K188A and K185E/K188E), of the zebrafish LPA_6_ receptor. However, none of these mutations had a large impact on the activity (S1 Fig). The effect of anchoring by K185 may be less critical than that of the activation for signal transduction, which makes it difficult to confirm the contribution of K185 by the assay method used in this study. Further analyses are required to clarify the role of K185 in the ligand anchoring.

**Fig 9:**
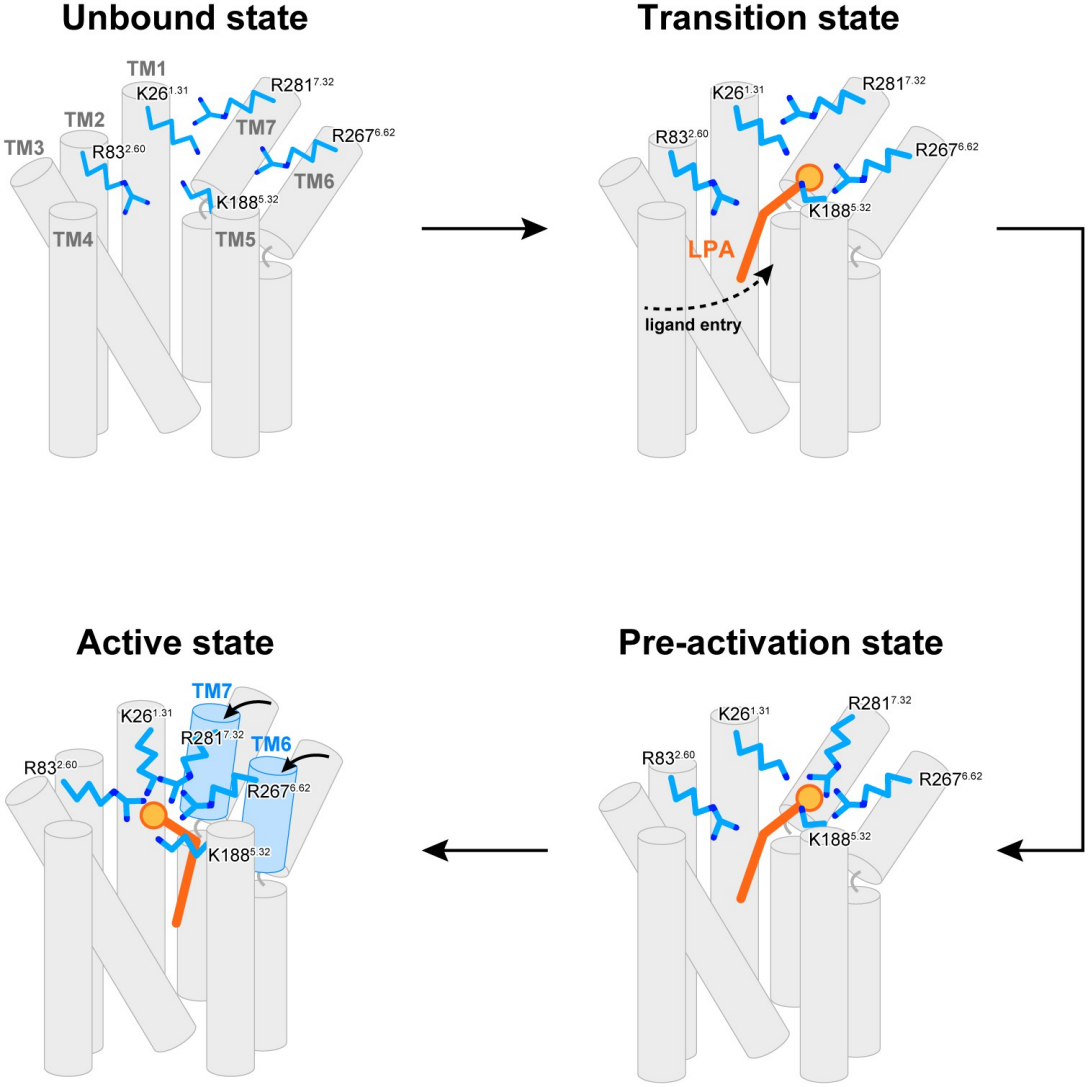
A schematic model of the LPA_6_ activation process proposed in this study.

In our previous study, the crystal structure of LPA_6_ and the structure-based functional analysis suggested that the functionally important basic residues (K26^1.31^, R83^2.60^, R267^6.62^, R281^7.32^) form the putative binding site for the ligand phosphate group. However, given the locations of these basic residues in the crystal structure and the size of the phosphate group, it is impossible for all of these residues to interact simultaneously with the ligand. Therefore, it has been hypothesized that some of these residues form the initial binding site for the ligand phosphate group in the pre-activation state, and then the large conformational changes of TM6 and 7 bring these residues near the ligand phosphate group to form the final binding site, thereby activating the downstream signaling pathway. This hypothetical model raises the question of which residues are involved in forming this initial binding site in the pre-activation state. In the present study, we performed two simulations based on Models 1 and 2, in which K26^1.31^, R83^2.60^ and K188^5.32^ (Model 1), and K26^1.31^, K188^5.32^, R267^6.62^ and R281^7.32^ (Model 2) are involved in the initial binding site formation. Despite the different initial binding manners of LPA, both simulations resulted in the similar binding mode of the LPA head group to Model 2; i.e., R267^6.62^ and R281^7.32^, but not R83^2.60^, are involved in the initial binding site (Fig 3; Macrostates 6 and 7). Therefore, we propose the updated model of the pre-activation state based on the present simulation (Fig 9), in which the initial binding site of the pre-activation state is formed by K188^5.32^, R267^6.62^, and R281^7.32^.

In conclusion, we reconstructed the ligand-binding process using MSM, based on the all-atom MD simulation with the 71.4 μs total length. The results provided detailed insights into the lateral access mechanism of the lipophilic ligand, including the transition state formation. In addition, the results suggested the recognition manner of the phosphate moiety of the ligand head group, by the initial binding site in the pre-activation state. In this study, we only focused on the process from the ligand-unbound to pre-activation state. We also ignored the effects of lipids other than POPC (i.e., cholesterol, etc.), which could have some impact on the ligand-binding and activation processes. Further functional, structural and computational analyses of the active state of LPA_6_, using a more realistic lipid system, will be required to further clarify the complete activation mechanism of LPA_6_.

## Methods

### Molecular dynamics simulation tool and force field

All MD simulations were performed using GROMACS ver. 5.0.7 [22] and the CHARMM36 [23] force field.

### System setup

For the initial structures of the simulation, three LPA_6_-LPA docking models (Models 1, 2 and 3) were used. All docking models were embedded within a 1-palmitoyl-2-oleoyl-*sn*-glycero-3-phosphocholine (POPC) bilayer, using the MemProtMD pipeline [24]. According to the original article, the N- and C-termini were capped with N-acetyl and N-methyl amide groups, respectively. Each simulation system was a 100 Å^3^ cube, solvated with 150 mM NaCl and TIP3 water molecules. The systems were equilibrated first for 0.1 ns under NVT conditions, with 10 kJ mol^-1^ Å^-2^ restraints for all heavy atoms (all atoms except hydrogen) of both the protein and ligand. Finally, the systems were equilibrated for 5.0 ns under NPT conditions with the same restraints, followed by a 100 ns equilibration without any restraints. For all of the following simulations, the LINCS algorithm was used for atom bond calculations. The system temperature was kept at 310 K by a Nosé-Hoover thermostat. Long-range electrostatic interactions were calculated by the particle mesh Ewald method.

### Steered MD simulation

To sample the initial structures for building MSM, steered MD simulations were conducted using the three equilibrated systems described above. The simulations were conducted under 10 kJ mol^-1^ Å^-2^ restraints for all C_α_ atoms of the protein, and the protein and the ligand. A 10 kJ mol^-1^ Å^-2^ harmonic potential was applied between their centers of mass, pulling the ligand off at a constant velocity of 0.4 Å/ns. The temperature was kept at 310 K, and the pressure was 1 bar. On two models, 5 simulations were performed for 50 ns, and coordinates for all atoms were sampled every 10 ps, to obtain 15,000 structures (= 2 models x 50 ns x 5 runs / 10 ps) in total. Finally, 714 structures (= 379 from Model 1 + 335 from Model 2) were randomly sampled from the above structures.

### Molecular dynamics simulation

All atoms of the sampled structures were assigned random velocities, and equilibrated for 500 ps under 10 kJ mol^-1^ Å^-2^ restraints for all C_α_ atoms of the protein and all atoms of the ligand. Using these initial structures, the main simulations were conducted for 100 ns without any restraints, and the coordinates for all atoms were sampled every 10 ps, to obtain 7,140,000 structures (= 714 initial structures×100 ns/10 ps) in total. The equilibration and the production run were both performed at 310K, 1 bar.

### Data analysis and visualization tools

We used MDtraj ver.1.8.0 [25] to handle the coordinates or trajectories and MSMBuilder ver. 3.8 [26] for featurization, clustering, building the MSM and reconstructing the pathway. We used MSMExplorer ver.1.1.0 [27] for the free energy calculation in the principle components space. All molecular graphics were illustrated with CueMol2 ver. 2.2.3.443 (http://www.cuemol.org/) and all movies were generated with VMD ver. 1.9.3 [28]. Plot graphics were generated with seaborn (https://seaborn.pydata.org/) and Matplotlib [29].

### Markov state model construction

All obtained structures were grouped into three datasets according to the original docking models. The MSM in each dataset was constructed by the following procedure. All structures were classified into 500 clusters with K-medoid clustering of the root-mean-square deviation (RMSD) values of all atoms, with the MiniBatchKMedoids module of MSMBuilder. The RMSD values were calculated for all atoms of the ligand and the 35 interacting residues: D23, K26, R83, V103, F106, Y107, M110, Y111, L115, G157, S158, T161, G162, L165, F179, E180, F182, S183, S184, K185, E186, W187, K188, L191, V195, I198, V201, G202, L260, Y263, R267, C274, E277, R281 and Y284. The interactions include hydrogen bond and hydrophobic interactions, and the range of the interaction distance was 1.5 Å–5.0 Å. Using all atoms, including these 37 residues and the ligand molecule, the RMSD values to these 500 reference structures were calculated with the LandMarkRMSDFeaturizer of MSMBuilder. These feature values were compressed into 10 dimensions by the principal component analysis (PCA) implemented in MSMBuilder. Structures were classified into 1,000 clusters by K-means clustering using 10 principal components (PC1–PC10), by the MiniBatchKMeans module of MSMBuilder. In that process, we transformed the MD trajectories into the 1,000 cluster transition trajectories.

The Markov state model (MSM) describes the dynamics of a system as a series of memoryless, probabilistic transitions between a set of states [15]. On the memoryless premise, the probability of the transition from state *i* to state *j* in a lag time of *τ* is described by a transition-probability matrix, **T**(*τ*) = {*T*_*ij*_(*τ*)}. For tuning the approximate lag time, the implied timescales, indicating how quickly the process reaches equilibrium, were calculated by the following formula:

$$t_{i}=-\;\frac{\tau }{ln\;\lambda_{i}}$$

where *t*_*i*_ indicates the *i*th slowest implied timescale, determined from the *i*th largest eigenvector of **T**(*τ*), *λ*_*i*_. In theory, if the implied timescales *t*_*i*_ converge as the lag time *τ* increases, then the model satisfies the Markov assumption [15]. In this study, the lag time was determined to be 10 ns after the tuning. Finally, the MSM was built based on the cluster transition trajectories by the MarkovStateModel module of MSMBuilder.

### Statistical analysis

Kinetically related microstates were grouped into 5 metastable states (macrostates) using PCCA^+ 20^. Ligand-unbound states were grouped into one macrostate. In this macrostate, the microstate with the largest population was chosen. The center of mass structure of this microstate was extracted using a *k*-d tree algorithm [30]. Using this structure as the starting state, we reconstructed a 100 μs (= lag time 10 ns x 10,000 steps) trajectory by the sample_msm module of MSMBuilder.

Transition path theory (TPT) is a way to extract the highest-flux pathways of the system from an estimated MSM [31]. To calculate the TPT path, we determined the final states by extracting the center of mass (using the *k*-d tree algorithm) of the largest cluster in the third dump on the PC1–PC3 energy map. TPT paths were generated by the TPT modules of MSMBuilder.

### Ligand-induced TGFα shedding assay

The ligand responses of the LPA_6_ mutants were determined by the TGFα shedding assay, which measures LPA_6_-mediated G12/13 signaling as described previously [5, 32]. Briefly, HEK293FT cells at a concentration of 2 x 10^5^ cells/ml were seeded in 4 ml of 10% FCS- and penicillin/streptomycin-containing DMEM (complete DMEM) per 6-cm culture dish and placed in a CO_2_ incubator at 37ºC for 1 day. A modified pEGFPC1 vector (Clontech) encoding the wild-type or a mutant LPA_6_, consisting of a haemagglutinin signal sequence, FLAG epitope tag and zebrafish LPA_6_ (residues 1–312) [5], was transfected together with a pCAGGS plasmid encoding alkaline phosphatase (AP)-tagged TGFα (AP-TGFα; human codon-optimized) into HEK293FT cells (seeded 1-day before the transfection in 4 ml per 6-cm culture dish at a cell concentration of 2 x 10^5^ cells/ml) by using a polyethylenimine (PEI) transfection reagent (400 ng LPA_6_ plasmid, 1 μg AP-TGFα plasmid, and 8 μl of 1 mg/ml PEI solution per 6-cm culture dish). After one day of culture, the transfected cells were harvested by trypsinization, neutralized with complete DMEM, washed once with Hank’s Balanced Salt Solution (HBSS) containing 5 mM HEPES (pH 7.4), and resuspended in 10 ml of HEPES-containing HBSS. The cell suspension was seeded at a volume of 80 μl (per well hereafter) in a 96-well culture plate (cell plate), in which 10 μl of 30 μM Ki16425 (Adooq), an antagonist for LPA1 and LPA3, was predispensed. After a 30-min incubation in a CO_2_ incubator, a 10 μl aliquot of serially diluted linoleoyl LPA (Echelon Biosciences; 10X, diluted in 0.01% bovine serum albumin (BSA) and 5 mM HEPES-containing HBSS) was added to the cells in triplicate and incubated for 1 h. The cell plate was then centrifuged, and 80 μl of conditioned media (CM) was transferred to an empty 96-well plate (CM plate). The AP reaction solution (80 μl, containing 10 mM p-nitrophenylphosphate (p-NPP), 120 mM Tris–HCl (pH 9.5), 40 mM NaCl, and 10 mM MgCl_2_) was dispensed into both the cell plates and the CM plates. The absorbance at 405 nm (A_405_) of the plates was measured with a microplate reader (SpectraMax 340 PC384, Molecular Devices), before and after a 1 h incubation at room temperature. Ligand-induced AP-TGFα release was calculated as described previously [32]. Unless otherwise noted, the background AP-TGFα release signals in the empty vector (mock)-transfected cells were subtracted from those in the LPA_6_-expressing cells. Using the Prism 8 software (GraphPad Prism), the LPA_6_-dependent AP-TGFα release signals were fitted to a four-parameter sigmoidal concentration-response curve, from which the EC_50_ and E_max_ values were obtained. The relative E_max_/EC_50_ value, also known as the relative intrinsic activity (RAi), a dimensionless parameter [33], was logarithmically transformed (Log RAi) and used to indicate receptor activity.

### Enzyme-induced TGFα shedding assay

The responses of the LPA_6_ mutants to PA-PLA1α were determined by a modified TGFα shedding assay, as reported previously [5]. In brief, HEK293FT cells in the growth phase were suspended in Opti-MEM I Reduced Serum Medium (Thermo Fisher Scientific) at a cell concentration of 4 x 10^5^ cells/ml, seeded in a 96-well plate (80 μl per well), and placed in a CO_2_ incubator. On the same day, the transfection solution (per well hereafter) was prepared by mixing 8 ng of the LPA_6_ plasmid, 20 ng of the AP-TGFα plasmid, and a titrated volume of the human PA-PLA1α pCAGGS plasmid (wild-type or the catalytically inactive S154A mutant), with 0.2 μl of 1 mg/ml PEI and 20 μl Opti-MEM I Reduced Serum Medium. The empty pCAGGS plasmid was used to balance the equal volumes of transfected plasmid. After adding the transfection solution (20 μl), the cells were cultured for 1 day. The 96-well plate was centrifuged at 190g for 2 min, and the supernatant (80 μl) was transferred to an empty 96-well plate. The AP activities in the cell plate and the CM plate were measured and calculated as described above, except the measurement interval was 15 min. The background AP-TGFα release signal in the absence of the PA-PLA1α plasmid was subtracted from those in the PA-PLA1α-expressing cells.

### Flow cytometry analysis

HEK293FT cells were seeded in a 12-well culture plate (1 ml per well) and transfected with plasmids (100 ng LPA_6_ plasmid and 250 ng AP-TGFα plasmid), as described above. One day after transfection, the cells were harvested with 0.53 mM EDTA-containing Dulbecco’s PBS (D-PBS). The cell suspension was transferred to a 96-well V-bottom plate and fluorescently labeled with an anti-FLAG epitope (DYKDDDDK) tag monoclonal antibody (Clone 1E6, FujiFilm Wako Pure Chemicals; 10 μg/ml diluted in 2% goat serum- and 2 mM EDTA-containing D-PBS (blocking buffer)) and a goat anti-mouse IgG secondary antibody conjugated with Alexa Fluor 488 (ThermoFisher Scientific; 10 μg/ml in diluted in blocking buffer). After washing with D-PBS, the cells were resuspended in 200 μl of 2 mM EDTA-containing D-PBS and filtered through a 40-μm filter. The fluorescent intensity of single cells was quantified by an EC800 flow cytometer equipped with a 488 nm laser (Sony). The fluorescent signal derived from Alexa Fluor 488 was recorded in the FL1 channel, and flow cytometry data were analyzed with the FlowJo software (FlowJo). Live cells were gated with a forward scatter (FS-Peak-Lin) cutoff of 390, setting a gain value of 1.7. Values of mean fluorescence intensity (MFI) from approximately 20,000 cells per sample were used for analysis.

## Supporting information

S1 Fig Analysis of the MSM construction by the bootstrap method. From the Model 1 trajectory set (379 trajectories), 300 trajectories (~80%) were sampled with replacement, and 5 datasets (Sets 1–5) were created. For each dataset, we constructed the MSM models and classified them into the macrostates, as described in the Methods section. The microstates of MSM are plotted on the PC1-PC3 plane. The sizes of the circles are proportional to the populations of the clusters, and the centers of the clusters are color-coded according to their macrostates, as in Fig 5a. The results gave similar distributions of the macrostates, showing the robustness of the MSM constructed in this study.
(PDF)

S2 Fig Similarity between the Model 1 and Model 2 simulation results. The histograms of the distances between the ligand head group (phosphate oxygen atoms) and the important basic residues (side-chain nitrogen atoms) of the receptor are plotted for the results of the Model 1 and Model 2 simulations. The resulting distributions overlap well, suggesting that the interactions and structural ensembles of the two simulations converged to a similar distribution, despite the differences in their initial docking poses.
(PDF)

S1 Movie Movie showing the reconstituted 100-μs trajectory of the LPA binding process. The ligand and conserved basic residues are shown in space-filling and stick models, respectively.
(MP4)

S1 File (DOCX)
