# Supplementary figures and images for: Lateral access mechanism of LPA receptor probed by molecular dynamics simulation
Source: PLoS One. 2022 Feb 3;17(2):e0263296. doi: 10.1371/journal.pone.0263296 (PMC8812926; doi:10.1371/journal.pone.0263296)

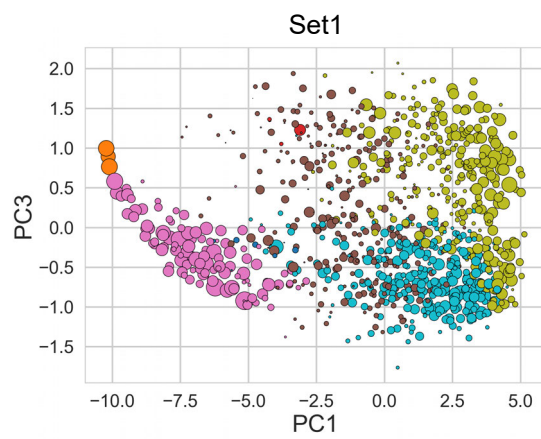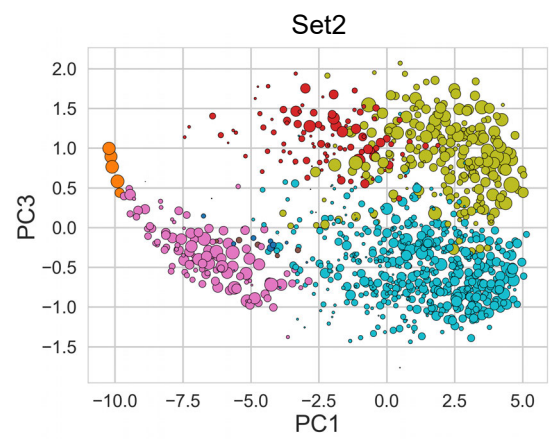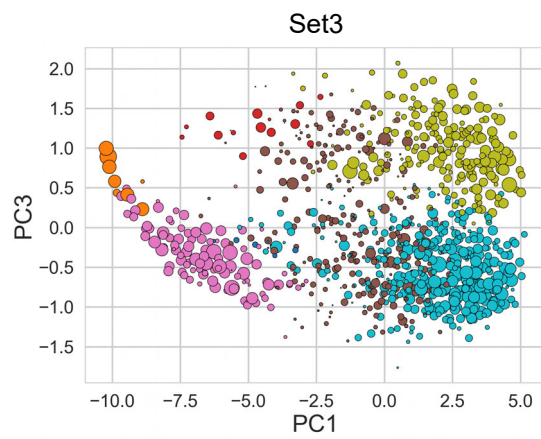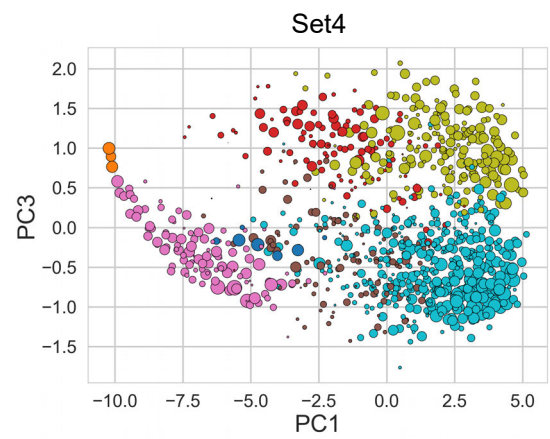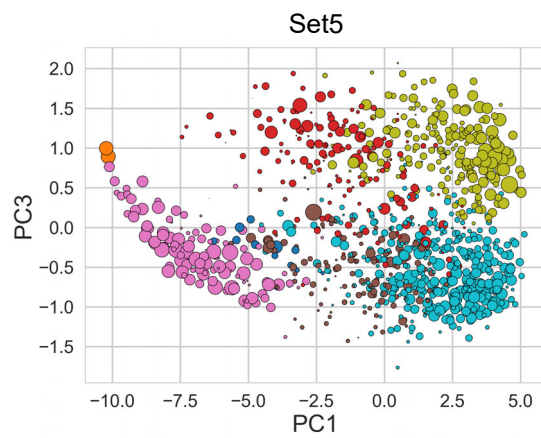

Suppl. Fig. 1

Supplement: S1 Fig — From the Model 1 trajectory set (379 trajectories), 300 trajectories (~80%) were sampled with replacement, and 5 datasets (Sets 1–5) were created. For each dataset, we constructed the MSM models and classified them into the macrostates, as described in the Methods section. The microstates of MSM are plotted on the PC1-PC3 plane. The sizes of the circles are proportional to the populations of the clusters, and the centers of the clusters are color-coded according to their macrostates, as in Fig 5a. The results gave similar distributions of the macrostates, showing the robustness of the MSM constructed in this study. (PDF) [file pone.0263296.s001.pdf]

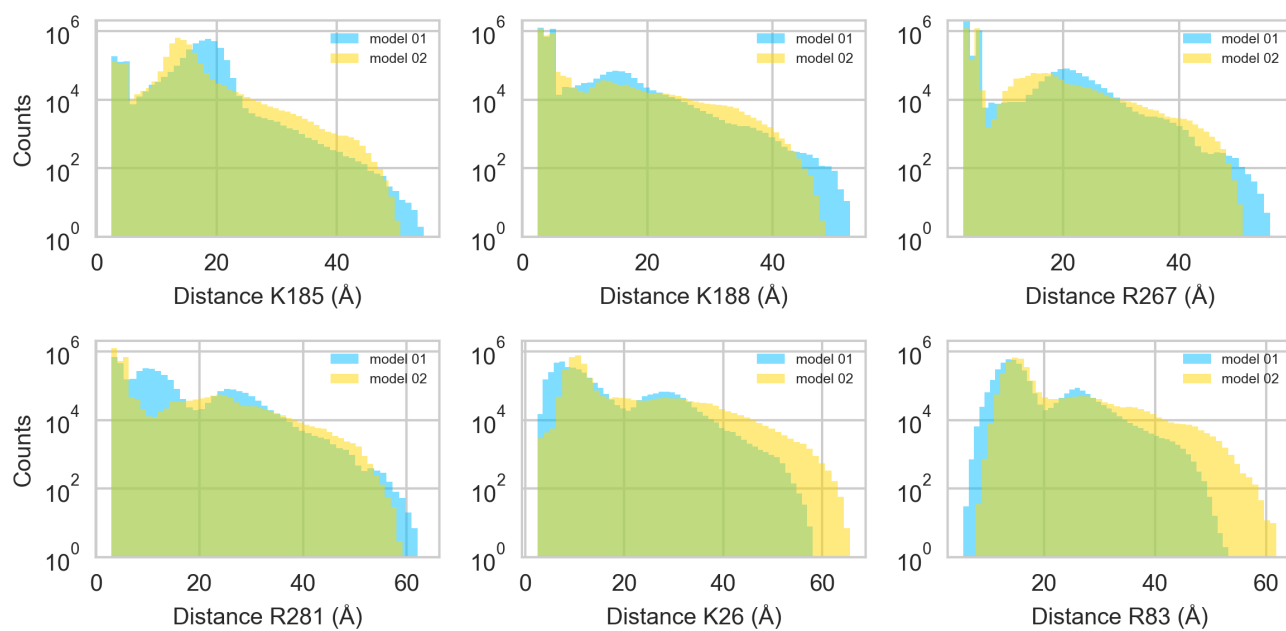

Suppl. Fig. 2

Supplement: S2 Fig — The histograms of the distances between the ligand head group (phosphate oxygen atoms) and the important basic residues (side-chain nitrogen atoms) of the receptor are plotted for the results of the Model 1 and Model 2 simulations. The resulting distributions overlap well, suggesting that the interactions and structural ensembles of the two simulations converged to a similar distribution, despite the differences in their initial docking poses. (PDF) [file pone.0263296.s002.pdf]
