# Supplementary material for: Lateral access mechanism of LPA receptor probed by molecular dynamics simulation
Source: PLoS One. 2022 Feb 3;17(2):e0263296. doi: 10.1371/journal.pone.0263296 (PMC8812926; doi:10.1371/journal.pone.0263296)
Supplement: S1 File — (DOCX) [file pone.0263296.s004.docx]

## Steered MD simulation

We obtained the sequential trajectory from the ligand-bound state to the unbound state by a “steered MD” simulation, in which the ligand was slowly separated from the receptor with external force. In the simulation, the harmonic potential was applied between the centers of mass of the ligand molecule and the protein, and the ligand was pulled parallel to the x-y (i.e., membrane) plane at constant velocity. During the simulation, the protein backbone was harmonically restrained to the initial structure. We performed this steered MD simulation five times each for Model 1 and Model 2 with different initial velocities. We obtained 379 and 335 initial structures from these pull-simulation trajectories of the Model 1 and Model 2 simulations, respectively, using random sampling.

## MSM building

To build the MSM for these simulations, we analyzed the MD simulation trajectories as follows (Fig. 2). At first, we classified all of the snapshot structures of the MD simulation into 500 clusters using the K-medoids clustering algorithm [1], based on the RMSD values of all atoms of the protein. We then chose the snapshot structures closest to the center of the clusters as the “reference structures.” These 500 reference structures are expected to represent unique reference points in the configuration space sampled by the 71.4 µs MD simulations. Next, we selected the 37 receptor residues that interact with the ligand atoms at distances shorter than 5.0 Å, and calculated the RMSD values to the 500 “reference structures,” using the non-hydrogen atoms of the selected receptor and ligand molecule residues. These RMSD values form vectors with 500 dimensions for each snapshot of the MD trajectories, and are expected to capture the features of the interactions between the ligand and receptor residues. These 500-dimensional feature vectors were further compressed into 10-dimensional vectors, using the principal component analysis (PCA) method. Subsequently, all snapshots of the MD trajectories were classified into 1,000 clusters, based on these 10-dimensional feature vectors, by K-means clustering. Finally, MSMs composed of 995 microstates were built from these clusters. The robustness of the analysis was verified using the bootstrap method (Suppl. Fig. 1).

## Principal component axes and ligand binding process

To further clarify the ligand binding mechanism from this energy landscape, we analyzed the correlation between these PCs and the ligand binding process. As shown in Fig. 4b, the “dissociated”, “partially-bound” and “bound” groups were aligned in the increasing direction of PC1, suggesting that PC1 is simply related to the ligand-receptor distance. To investigate this notion, we compared the PC1 values with the distances between the ligand head group and the basic amino acid residues, which are reportedly important for the ligand binding (Fig. 4c). The results showed that PC1 is highly correlated with these distances between the ligand and receptor residues, and the two stable states in the energy landscape correspond to the ligand-bound and -unbound states.

We next analyzed the relationship between PC2 and the ligand and receptor conformations. We plotted the initial structures obtained by the steered MD simulation on the PC1-2 plane (Fig. 4b, left panel, dark blue plot), and found that the initial structures are clustered around the high-PC2 region. These initial structures are biased by the pulling forces of the umbrella potential, and thus the PC2 axis may be related to the relaxation process to the unbiased states. To corroborate this notion, we investigated the ligand conformations in the initial structures with high PC2 values. The conformation of the ligand is evaluated as the “z” value, calculated as the cosine of the angle between the normal vector of the membrane plane and the vector connecting the ligand’s phosphorus atom to the 12th carbon atom (Fig. 4d). The results showed that the acyl chain of the ligand in these initial structures assumed a horizontal orientation to the membrane plane (Fig. 4d), which gradually relaxed to form the vertical orientations during the 100-ns simulation without biasing potential. To further analyze the relationship between PC2 and ligand conformation, we compared the “z” and PC2 values (Fig. 4e). The results revealed that PC2 is highly correlated with the direction of the acyl chain, with large PC2 values corresponding to the horizontal orientation. In the initial structures, the weak interactions between the ligand acyl chain and the hydrophobic residues of the receptor may be easily disrupted by the biasing potentials, as compared to the strong interactions between the ligand head group and the basic residues of the receptor, thus finally resulting in the horizontal orientation of the ligand acyl chain. During the simulation of the production runs without biasing potentials, the initial structures with high PC2 values were relaxed to the low PC2 conformation with a vertical orientation. Furthermore, the low free-energy basins around the low PC2 regions suggested that the sampling of the relaxed conformations was sufficient to provide an unbiased view of the ligand binding dynamics.

As noted above, the interaction between the ligand head group and the R281^7.32^ side chain is the major difference between Macrostates 6 and 7. Further visual inspection of representative snapshots of Macrostates 6 and 7 also suggested that the PC3 value may be related to the interaction between the head group and R281^7.32^ (Fig. 3b). In the bound group, the distance between the ligand head group and R281^7.32^ correlated well with the PC3 value (Fig. 5b). The transition between the high- and low-PC3 basins of the bound group (Fig. 5a, right panel) may correspond to the process for deeply accommodating the ligand head group within the basic pocket formed around R281^7.32^.

1. de Hoon, M. J. L., Imoto, S., Nolan, J. & Miyano, S. Open source clustering software. *Bioinformatics* **20**, 1453–1454 (2004).
